# Location, location, location: the BRMS1 protein and melanoma progression

**DOI:** 10.1186/PREACCEPT-2032291833666638

**Published:** 2012-02-22

**Authors:** Adam I Riker, Rajeev S Samant

**Affiliations:** 1 Advocate Christ Medical Center, Advocate Cancer Institute, Oak Lawn, IL, USA; 2 Department Oncologic Sciences, Mitchell Cancer Institute, University South Alabama, Mobile, AL, USA

**Keywords:** BRMS1, melanoma, metastasis, tumor suppressor

## Abstract

The metastasis suppressor, BRMS1, has been demonstrated to cause dramatic regression of metastatic lesions without blocking orthotopic tumor growth. The role of BRMS1 is well-documented for several non-melanoma malignancies, such as breast cancer, ovarian cancer and non-small-cell lung cancer. However, its role in melanoma is just beginning to be understood, with a recent article by Slipicevic *et al. *highlighting the levels of expression of BRMS1 in benign nevi, primary and metastatic melanoma samples. Their findings emphasize that the intracellular location of BRMS1 protein (cytoplasmic or nuclear), appears to have a significant impact upon the metastatic capacity of melanoma cells. Interestingly, this selective localization translates into a statistically significant decrease in the relapse-free period in melanoma patients, further associated with a thicker Breslow's depth of primary melanomas. However, and more importantly, this study begins to define a clearer role for BRMS1 in melanoma that is strictly dependent upon its cellular location, with nuclear expression associated with invasive and metastatic capacity and cytoplasmic expression resulting in repressive effects upon progression and metastasis.

Please see related article: http://www.biomedcentral.com/1471-2407/12/73

## Background

It has been over a decade since the initial reports of the discovery and functional relevance of Breast Cancer Metastasis Suppressor 1 (BRMS1) [1,2]. Though the initial reports stemmed from research performed on breast cancer, convincing independent studies on suppression of melanoma metastasis by BRMS1 were described very early [3]. However, as the molecular complexity of mechanisms of action of BRMS1 became evident through several elegant studies [4-11], defining the exact physiological and clinical context of these activities remains elusive. Lastly, the central question remains: Does the loss of BRMS1 expression result in disease progression?

One well-described scenario may entail the loss of BMRS1 expression in tumor tissue secondary to epigenetic silencing [12,13]. Other possibilities include changes in expression related to BRMS1 regulation at the transcriptional and post-translational level, further complicated by potential changes in overall protein stability. In a recent study published in *BMC Cancer *by Slipicevic *et al*., the authors show that the intracellular location of BRMS1, whether cytoplasmic or nuclear, appears to affect relevant outcome parameters in melanoma patients.

### Understanding why location is important

**Figure 1:**
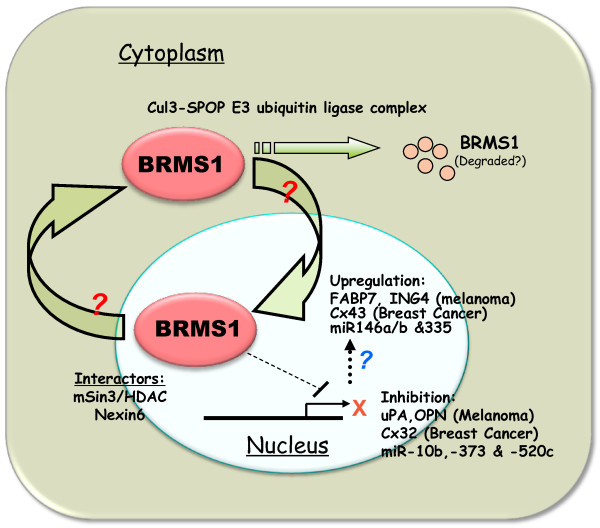
**Location(s) of BRMS1 and corresponding activities**. BRMS1 is a transcription co-repressor and can shuttle between nucleus and cytoplasm. BRMS1 levels are governed by ubiquitin-mediated degradation. In the nucleus, it shows inhibition of some transcripts and up-regulation others. However, some of these regulations may be due to its cytoplasmic activities (such as deacetylation of NFκB members). The ultimate impact of BRMS1 on a specific tumor cell appears to be tumor type and context-dependent. Abbreviations: BRMS1, breast cancer metastasis suppressor-1; FABP7, fatty acid binding protein-7; ING-4, inhibitor of growth family, member-4; Cul3, Cullin-3; SPOP, speckle-type POZ domain protein; mSin3, mammalian Sin3; HDAC, histone deacetylase; Cx32, 43, connexin-32, -43; uPA, urokinase-type plasminogen activator; OPN, osteopontin; miR, micro ribonucleic acid; Nexin6, sorting nexin-6.

It is well known that the intracellular location and timing of protein expression could manifest as diverse biological activities. For example, the tumor suppressor gene, P53, has been reported to be actively expressed in the cytoplasmic, mitochondrial, nuclear and nucleolar regions of tumor cells [14,15], with distinct activities dependent upon its localization. BRMS1 is mainly considered to be a nuclear transcription co-repressor, due to the presence of definitive nuclear localization signals and reports of its participation in the chromatin remodeling complex(es). Interestingly, BRMS1 has a functional nuclear export signal indicating its capability to shuttle through cellular compartments [16] (Figure 1). However, activities of BRMS1 within the cytosolic compartment are still to be defined. Slipicevic *et al. *also show that nuclear presence of BRMS1 in melanoma correlates with fatty acid binding protein 7 (FABP7). This study implies a possible role of nuclear BRMS1 as a promoter of invasion, further supported by silencing of BRMS1. Further relevant data may be obtained through distinct experiments that target BRMS1 function to distinct compartments, revealing more mechanistic details. It will also be interesting if FABP7 is found to be a downstream target of nuclear BRMS1.

### Does it really matter where BRMS1 expression is found?

This paper has consistently shown that there are significant differences in staining intensity between cytoplasmic and nuclear BRMS1 expression. Ultimately, the question can be asked as to whether the specific location of BRMS1 activity can make a difference in the destined outcome for melanoma patients. The work shows that high intensity cytoplasmic staining for BRMS1 expression is associated with a significantly higher relapse-free period compared to lower staining cells. Conversely, intense nuclear staining is associated with a lower relapse-free period compared to higher staining cells within the nucleus of melanoma cells.

Ultimately, does it really matter where BRMS1 is being expressed to the patient or clinician? Is there anything that can be done to change the ultimate outcome for these patients? Can one utilize this ratio of BRMS1 staining information as a potential prognostic marker for such patients? BRMS1 can have multiple spliced variants [17]; thus, is the location specific to any of these splice variants? Can we change the balance in favor of higher expressing cytoplasmic BRMS1 expression, with concomitant lower nuclear levels? What are the details of interactions between FABP7, activated ERK1/2 and Akt within the nucleus? A recent study has shown that the cell cycle regulator, ING4, is a suppressor of melanoma angiogenesis, regulated and induced by BRMS1 expression and further inhibiting NF-KB activity and IL-6 expression [18]. Thus, many other questions emerge unanswered and will require further research to elucidate the importance of BRMS1 expression in melanoma, whether nuclear or cytoplasmic.

Recent evidence strongly suggests that BRMS1 represents an important biomarker with prognostic significance, further serving as a therapeutic target for melanoma patients [19]. Other correlations and possible biomarkers have also been identified, such as an inverse relationship with osteopontin levels in melanoma cells and tumor specimens [13]. Looking forward, if one was capable of restoring BRMS1 expression via reversal of epigenetic silencing, could we concomitantly regulate its intracellular location and activity in tumor cells to achieve the desired therapeutic outcome in melanoma patients.

## Conclusion

Slipicevic *et al. *show for the first time that there is a differential expression of BRMS1 within the cytoplasm and nucleus of melanoma cells, with cellular localization determining its effect within the tumor microenvironment. Such differences are associated with a significant difference in the relapse-free period, with cytoplasmic BRMS1 expression restricting melanoma progression and nuclear expression likely associated with cell invasion. From a treatment perspective, the importance of this study will be realized if bioactive agents can be developed that have the capacity to change both the (natural) balance and location of BRMS1 expression within melanoma cells.

## Abbreviations

BRMS1: Breast Cancer Metastasis Suppressor-1; ERK-1: -2: Extracellularly-regulated Kinase-1: -2; FABP7: Fatty Acid Binding Protein-7; IL-6: Interleukin-6; ING-4: Inhibitor of Growth Family, member-4; NF-ΚB: nuclear factor KB.

## Competing interests

The authors declare that they have no competing interests.

## Authors' contributions

AIR and RSS contributed equally to this commentary. Both authors were involved in the development, writing and revisions of this manuscript.

## Authors' information

Adam Riker is the Medical Director of the melanoma program at Advocate Christ Medical Center and Cancer Institute in Oak Lawn, Illinois. Rajeev Samant is an associate professor within the Department of Oncologic Sciences and is the Head of the Cellular and Biomolecular Imaging Facility at the University of South Alabama, Mitchell Cancer Institute in Mobile, Alabama.

## Pre-publication history

The pre-publication history for this paper can be accessed here:

http://www.biomedcentral.com/1741-7015/10/19/prepub
